# Experimental investigation of the hydraulic and heat-transfer properties of artificially fractured granite

**DOI:** 10.1038/srep39882

**Published:** 2017-01-05

**Authors:** Jin Luo, Yongqiang Zhu, Qinghai Guo, Long Tan, Yaqin Zhuang, Mingliang Liu, Canhai Zhang, Wei Xiang, Joachim Rohn

**Affiliations:** 1 China University of Geosciences (Wuhan), Faculty of Engineering & School of Environmental Studies, Wuhan 430074, P.R. China; 2 Huanghe Hydropower Development CO., LTD., State Power Investment Corporation, 810008 Xining, Qinghai, P.R. China; 3 University of Erlangen-Nürnberg, Geo-Center of Northern Bavaria, Schlossgarten 5, 91054 Erlangen, Germany

## Abstract

In this paper, the hydraulic and heat-transfer properties of two sets of artificially fractured granite samples are investigated. First, the morphological information is determined using 3D modelling technology. The area ratio is used to describe the roughness of the fracture surface. Second, the hydraulic properties of fractured granite are tested by exposing samples to different confining pressures and temperatures. The results show that the hydraulic properties of the fractures are affected mainly by the area ratio, with a larger area ratio producing a larger fracture aperture and higher hydraulic conductivity. Both the hydraulic apertureand the hydraulic conductivity decrease with an increase in the confining pressure. Furthermore, the fracture aperture decreases with increasing rock temperature, but the hydraulic conductivity increases owing to a reduction of the viscosity of the fluid flowing through. Finally, the heat-transfer efficiency of the samples under coupled hydro-thermal-mechanical conditions is analysed and discussed.

Geothermal energy has been attracting more and more attention in the past few decades due to its advantageous characteristics of cleanness, renewability and environmental friendliness^1^. Middle enthalpy geothermal energy, which occurs at temperatures varying from 90 to 150 °C, is generally stored in sandstone, limestone or granite at a depth of thousands of metres^2^. It is commonly used for municipal heating or electricity power generation. Geothermal energy is extracted by circulating cold water through hot rock, which leads to the exchange of heat^3^. Deep rock beds often have very low permeability (e.g., granite) or are without natural groundwater flow. Within deep geothermal systems (>400 m), fluid flow through the rock is essential to the thermal efficiency of geothermal projects. To enhance energy efficiency, hydraulic stimulation is commonly carried out to create artificial fractures and thus improve the hydraulic conductivity of rock^4^. The stimulation effect is generally evaluated by considering the hydraulic properties of the fractured rock^5^.

Many previous experimental studies have been conducted to investigate the hydraulic properties of fractured rock under coupled hydro-mechanical conditions^6^,^7^,^8^. Brace *et al*.^9^ conducted a study to investigate the permeability of a single fracture in granite. The results indicated that the hydraulic conductivity of fractures decreased with an increase in the confining pressure at room temperature. Xi *et al*.^10^ examined the effect of thermal stress of seepage in a fractured rock mass, and it was found that the fracture gradually closed with increasing rock temperatures owing to the effects of thermal expansion. Wang *et al*.^11^ analysed the temperature effects of underground disposal of nuclear waste on the surrounding granite rock. The findings clearly showed that compression stress occurred around the disposal cave as a result of the high temperature induced by nuclear radiation.

Concerning hydraulic tests at high temperatures, Zhu and Guo simulated the flow characteristics of a nuclear waste deposit in laboratory tests^12^. The temperature of the heat source was controlled between 200 °C and 300 °C, and the results showed that the permeability of the rock decreased with an increase in rock temperature. Barnabe^13^ measured three samples of Chelmsford granite cored in mutually perpendicular directions. The data suggested a decrease in the coefficient with increasing confining pressure. Morrow *et al*.^14^ conducted a complete granite and fractured granite seepage test under high-temperature conditions (300–500 °C). The results showed that the conductivity decreased with time and that the fracture closed rapidly under high-temperature conditions. The permeability of the fractured granite was reduced to that of intact granite after 20 days of testing.

Furthermore, some studies have been conducted to examine the chemical processes that occur during fluid flow through rock fractures. Yasuhara *et al*.^15^ investigated seepage in the fissures of homogeneous quartzite exposed to an effective stress of 1.4 MPa and a temperature of 20–120 °C. The test duration was 3150 h, during which the size of the aperture decreased from 18.5 μm to 7.5 μm, and then increased to 13 μm. The concentration of dissolved minerals increased during the test. Caulk *et al*.^16^ experimentally observed the evolution of the fracture aperture within a granite-based Enhanced Geothermal System (EGS) in an attempt to better understand reductions in fracture permeability. It was found that mechanical effects had a greater effect on aperture change than the effluent chemistry.

Theoretical and numerical researches have also been conducted to investigate the hydraulic properties of fractured rocks with attention to fracture morphology^17^,^18^,^19^. Kranz *et al*.^20^ proposed an empirical formula for evaluating the permeability of a jointed rock mass. Abdallah *et al*.^21^ studied the coupling of water and heat exchange in a jointed rock mass using the discrete element method. The research indicated that fluid convection is sensitive to the size of the fracture aperture, the circulation velocity and the viscosity of the fluid flowing through. Chai and Han^22^ developed a continuous-medium model for seepage-temperature coupling in a rock mass. A finite elemental solution was proposed, and the interaction between temperature and seepage in fractured rocks was discussed. Recent progress in the study of fractured rock masses has come from an international collaboration called Development of Coupled Models and their Validation against Experiments (DECOVALEX)^23^. The DECOVALEX project was initiated in 1992 and aims to advance the understanding and modelling of coupled thermo-hydro-mechanical (THM) and thermo-hydro-mechanical-chemical (THMC) processes in geological systems. After nearly 20 years of research, it has been shown the fractures could be opened or closed by external stress and fluid pressure. The stress field, temperature field and seepage field interact with each other in a rock mass, and so a rock mass should be considered as a system.

The studies described above investigated the hydraulic properties of fractured rocks by considering different coupling conditions^9^,^10^,^11^,^12^,^21^,^22^,^23^,^24^,^25^,^26^. However, there remains a lack of data about the morphology of fractures. To quantitatively analyse the hydraulic properties of fractured rocks, morphological information about the fractures is indispensable. By considering these existing problems, this paper aims to investigate the hydraulic properties of six fractured granite samples with a focus on to the surface conditions of the fractures. The materials and study methodology are described in the next section. First, a three-dimensional model was built, and the surface morphology of the fractures was then determined. The results were then analysed and are discussed below. Hydraulic properties and heat-transfer efficiency were analysed in relation to the confining pressure and rock temperature. The findings of this study are presented in the final section.

## Materials and study methodology

### Description of the samples

The study area is located in the Gonghe-Guide basin in Qinghai Province and lies between the Kunlun-Qinling Mountains and the Hexi structural belt. This area is situated in northwest China. The Gonghe-Guide basin is a Cenozoic fault basin that is estimated to have good geothermal potential. All the rock samples in the present work were collected from the northern part of the study area. In 2013, a geological survey was completed by the Hydrogeology, Engineering and Environmental Geological Survey Institute of Qinghai Province and China University of Geosciences (Wuhan). A geological survey and drilling indicated a Hot Dry Rocks (HDR) reservoir that has an area of 150 km^2^ in the northern basin. The rock temperature was greater than 150 °C at a depth of over 2000 m. This was the first confirmation of such a large-scale HDR reservoir in China.

In this study, the physical parameters of the prepared rock samples were first measured in the laboratory. The density of the samples was tested using the wax-coating method, and thermo-physical properties were determined using an ISOMET 2114 thermal conductivity instrument manufactured by Applied Precision Ltd. (Bratislava, Slovakia). The instrument can achieve an accuracy of ±5.0% for the measurement of thermal conductivity. During the thermo-physical property tests, the surfaces of the rock samples were cut and polished to meet the measuring requirements. Thermo-physical information about the rock samples that were collected from the study area is listed in Table 1; sample set number one (#1, #2 and #3) was drilled from one site, and set number two (#4, # 5 and #6) was collected from another site.

### Preparation of the samples

**Figure 1:**
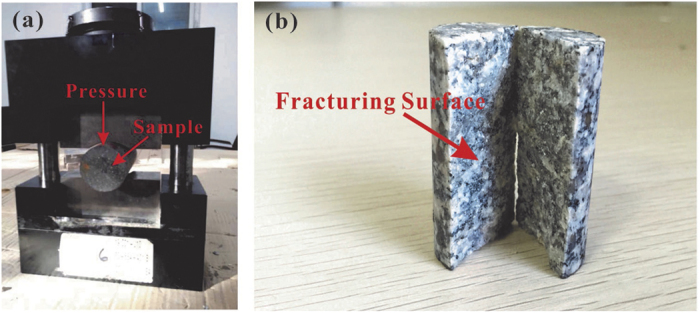
Improved Brazilian testing setups (**a**) and the sample after improved Brazilian test, the sample is with diameter of 25 mm and length of 40 mm (**b**).

Artificial fractures were created in the rock samples using the improved Brazilian testing method and the rock Mechanics Testing System (MTS). A metal fixture was used to hold the samples and a pressure force was applied to vertically displace the sample with a speed of 3 × 10^−3^ mm/s, as shown in Fig. 1a. Hence, fractures were created by the tension force that was transferred to the fixture by the compression force. All the samples are cylindrically shaped with a diameter of 25 mm and a height of 40 mm. In the present work, a single fracture with a rough surface was created; the single fracture ran through each sample in the axial direction, as shown in Fig. 1b.

### Morphological information about the fractures

**Figure 2:**
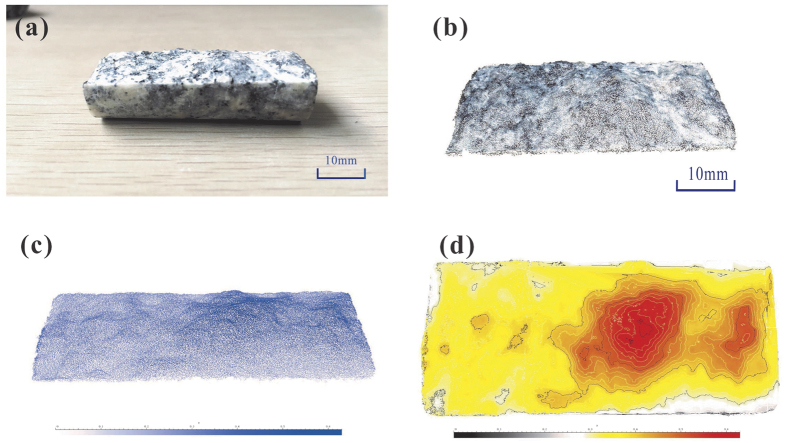
Building of three-dimensional model of the fractures, (**a**) the fractures of rock sample, (**b**) Point clouds of fractures, (**c**) Schematic diagram of 3D model, (**d**) Height contour diagram of 3D model.

To determine the morphology of the fractures in the rock samples, three-dimensional models were built. The process for establishing the 3D model can be simply divided into 4 stages: photographing, extracting the point cloud, denoting abnormal points, and generating the 3D digital model. First, the surface of the fractured rock sample was photographed to collect the morphological information. Second, the point cloud, which includes 1,300,000 points with coordinate information, was extracted based on the photographs.The high resolution of the adjacent point can reach up to 0.01 mm. Third, setting the threshold of each direction to eliminate abnormal points; a diagram of the corrected point cloud is shown in Fig. 2c. Finally, the 3D model of the fractures and the height contour line was created using the point matrix. In Fig. 2d, the colour indicates the fluctuation of the point height of the fractures. The warmer the colour, the greater the height of the point.

### Experimental setup

#### Sample instrumentation

**Figure 3:**
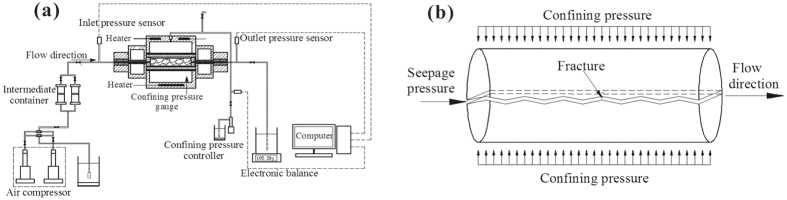
Schematic diagram of the testing instrument (**a**) and sketch of the testing setup of the fractured granite samples (**b**).

In the present work, the testing system consisted of an air compressor, water container, heating oven, gripper, electronic scale, confining pressure controller and computer, as shown in Fig. 3a. Within the testing system, the hydraulic properties of the fractured samples under coupled hydro-thermal-mechanical conditions can be studied. The instrument can apply hydraulic pressures varying from 0 to 32 MPa and confining pressures ranging from 0 to 32 MPa with an accuracy of ±0.25%. The rock temperature can reach a maximum of 150 °C with an accuracy of ±0.5 °C. The diameter of the samples is limited to a maximum of 25 mm with this instrument. All the data, including the confining pressure, fluid temperature and flow rate, were automatically recorded with a data acquisition system.

#### Testing process

To study the hydraulic properties and the heat-transfer rate of fractured granite samples under coupled hydro-thermal-mechanical conditions, two sets of samples were prepared in triplicate. Sample set number one (#1, #2 and #3) was used to investigate the hydraulic properties under different confining pressures, and sample set number two (#4, #5 and #6) was tested with different temperatures, as shown in Fig. 3b. All the tests were performed taking the 3D morphology of the fractures into consideration. The procedure for the tests is listed as follows:

(1) A single fracture was created in each sample using the Brazilian splitting method. The fractures ran through the whole sample parallel to the axial direction of the granite specimens.

(2) The samples were loaded into the testing instrument. The temperature of the heating oven and the confining pressure were set. The hydraulic pressure was then increased and the hydraulic properties were measured by recording data including flow rate, temperature, confining pressure and seepage pressure during the testing process. Data are shown in Fig. 3b. The procedure ensured that the fracture was saturated by placing the instrument vertically and pumping the fluid through the sample from the bottom to the top to force air out.

(3) For sample set one (#1, #2 and #3), the temperature of the heating oven and the hydraulic pressure were fixed. The hydraulic properties of the samples were investigated by exposing the samples to different confining pressures (There are 4 MPa, 6 MPa, 8 MPa, 10 MPa, 12 MPa, 14 MPa, 16 MPa, 18 MPa, 20 MPa, 22 MPa and 24 MPa). To remain a steady confining pressure and temperature, 30 minutes are waited before adapting the fluid flow. Afterwards, the flowing tests were conducted with duration of 60 minutes.

(4) For sample set two (#4, #5 and #6), the hydraulic pressure and the confining pressure were fixed. The hydraulic properties of the samples were investigated as the rock temperature increased from 25 °C to 100 °C in increments of 5 °C.

(5) During the experiments, data including inlet and outlet flow rate, temperature and pressure were continuously gathered by a data logger.

#### Testing data treatment

For rock samples with a single fracture, hydraulic conductivity can be determined by the fracture aperture^27^,^28^,^29^. In this work, the size of the hydraulic aperture of each fracture was calculated by following the cubic law for fluid flow in a single-fracture rock mass, which was proposed by Soviet researchers^30^. By considering the fluid viscosity, fluid flow rate and hydraulic gradient, the fracture aperture can be expressed as follows:


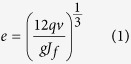


where 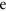 represents the size of the fracture aperture (m), 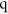 represents the flow rate per unit width of the fracture (m^2^/s), 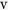 represents the kinematic viscosity coefficient of fluid (g/m^2.^s^2^), 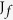 represents the hydraulic gradient in the fracture (-), and 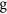 represents the acceleration of gravity (m/s^2^).

Based on Equation (1), the hydraulic conductivity coefficient can be further determined according to the following equation:


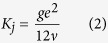


where 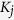 represents the hydraulic conductivity^31^,^32^.

The heat-transfer rate of the seepage in the fractured granite wass calculated using the recorded inlet and outlet fluid temperatures and the flow rate. It can be expressed as follows:


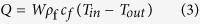


where 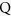 represents the specific thermal exchange rate (J/s), 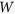
represents the volumetric flow rate of the heat carrier fluid (m^3^/s), 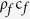 represents the heat capacity of the heat carrier fluid (J/m^3^·K), 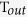 represents the outlet fluid temperature for the heat carrier fluid (°C), and 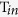 represents the inlet fluid temperature (°C)^33^,^34^.

## Results and Discussion

### Morphological information and hydraulic properties

**Figure 4:**
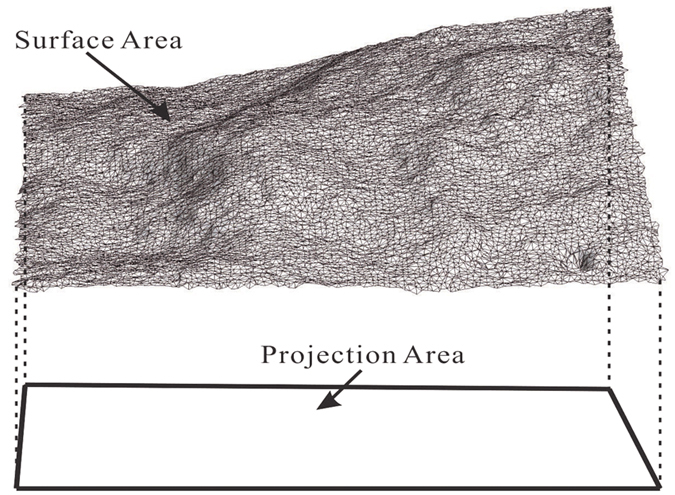
Schematic diagram of area ratio of fractures.

To describe the roughness of the surface of fractures, the area ratio was calculated using the surface area to divide the projected area in the X-Y plane of the fractures, as shown in Fig. 4. The area ratio is used to describe the relative roughness of fracture. In general, the larger the value of the area ratio, the rougher the fracture surface. The results listed in Table 2 show that the area ratio varies from 2.045 to 4.394, indicating different degrees of roughness for the fractures of the six samples. Sample #1 was the least rough, and Sample #4 was the roughest. The morphological information was further used for analysis of the hydraulic properties of the samples.

**Figure 5:**
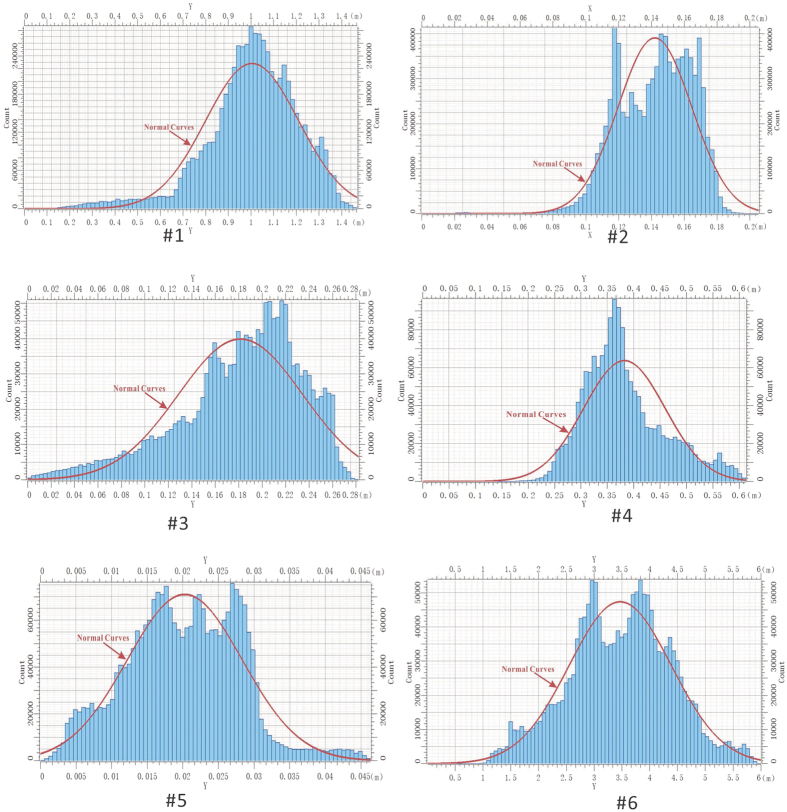
Frequency diagram of height distribution of fractures of the six prepared samples.

More specifically, the frequency of the point distribution with different heights was analysed. The height frequency is shown in Fig. 5. Furthermore, the maximum and secondary height distribution frequency was investigated, as listed in Table 3. The results show that Sample #4 has the maximum frequency value of surface height distribution, and Sample #5 has the minimum value. A large frequency value of means reduced fracture roughness. The maximum fluctuation indicates that Sample #5 had the highest degree of surface roughness, followed by Sample #6. Sample #4 had the lowest degree of roughness.

Table 4 displays the hydraulic apertures and conductivities of the rock samples exposed to a confining pressure of 4.0 MPa and a rock temperature of 25 °C. The results show that Sample #3 had the largest fracture aperture (425 μm), followed by sample #2 (280 μm) and Sample #1 (180 μm). By taking the 3D morphological characteristics of the fractures into account, the fracture aperture size was found to be strongly related to the area ratio. A larger area ratio gives rise to a larger aperture in the fractured samples, as shown in Tables 2 and 4. The area ratio correlates with the roughness of the fractures in the samples. Here, a higher surface roughness indicates a larger fracture aperture. Conversely, analysis of the height distribution frequency showed that Sample #3 had the lowest frequency, indicating the roughest morphology. Samples #1 and #2 were estimated to have a similar maximum frequency.

Furthermore, the hydraulic conductivity was investigated, and we found trends similar to those of the aperture. Sample #3 had the largest hydraulic conductivity (16 cm/s), followed by Sample #2 (7 cm/s) and Sample #1 (3 cm/s). The results show that the hydraulic conductivity is proportional to the fracture aperture. These findings indicate that the roughness of fractures has a significant influence on the hydraulic properties and that the area ratio is useful in the evaluation of fracture roughness.

### Effect of confining pressure

**Figure 6:**
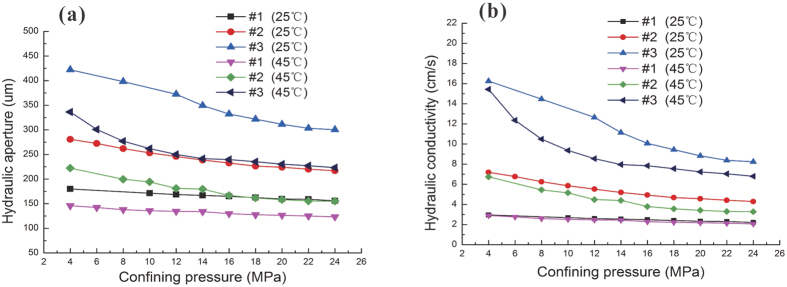
Relationship between confining pressure and fracture aperture with the hydraulic pressure is 0.9 MPa (**a**) and relationship between confining pressure and hydraulic conductivity with the hydraulic pressure is 0.9 MPa (**b**).

Many theoretical and experimental studies have shown that the physical and hydraulic properties of rock can change in response to the coupling of stress and temperature conditions^35^,^36^. To investigate the effects of confining pressure on hydraulic properties, the temperature and the hydraulic pressure were fixed at constant values. Hydraulic parameters such as fracture aperture size and hydraulic conductivity were investigated by increasing the confining pressure. Figure 6a shows the fracture aperture size decreasing with an increase in confining pressure. Large-scale fractures are less affected by stress, while small-sized fractures are strongly affected by stresses^37^. The aperture tends to be constant as the confining pressure increases to 18.0 MPa. Therefore, it can be considered that the fracture size is relatively large. The change at 18 MPa is due to the stiffness of the fracture, resulting in mechanical deformation. As a previous study indicated, increasing the confining pressure by coupling of the stress-temperature seepage leads to closing of fractures^38^. Hence, the decreasing size of the aperture can be attributed to compression of the fracture. The closing of the aperture is very easy by adapting a confining pressure and becomes more difficult when the two sides of the fracture surface have a large area of contact. Hence, the size of the apertures tends toward a constant value with increasing confining pressure.

Furthermore, hydraulic conductivity was determined using Equation (2), and the results are shown in Fig. 6b. Similar to fracture aperture, hydraulic conductivity was also found to decrease with increasing confining pressure. Hydraulic conductivity depends mainly on the fracture aperture size, and hence a similar decreasing trend was observed between the hydraulic conductivity and the confining pressure.

However, a different decreasing trend for hydraulic conductivity was shown among three of the samples that were investigated. Sample #3 had a decreasing trend for hydraulic conductivity, and Sample #1 had a slightly decreasing trend. The fractures of different rock samples had different morphological roughnesses, as shown in Table 2. A larger area ratio corresponded to a rougher surface and more of a decreasing trend for both the fracture aperture and the hydraulic conductivity. This finding indicates that hydraulic properties are sensitive to the roughness of fractures.

### Analysis of temperature effects

To examine temperature effects, permeability tests were conducted by exposing the samples to different temperatures. Sample set number one (#1, #2 and #3) was damaged somehow after a long period of testing at a high confining pressure. These samples were then not suitable for use in further experiments. Hence, sample set number two (#4, #5 and #6) was used. During the tests, the inlet fluid temperature was kept constant at 20 °C, and the outlet fluid temperature was measured at different rock temperatures. The confining pressure remained constant during the testing process.

**Figure 7:**
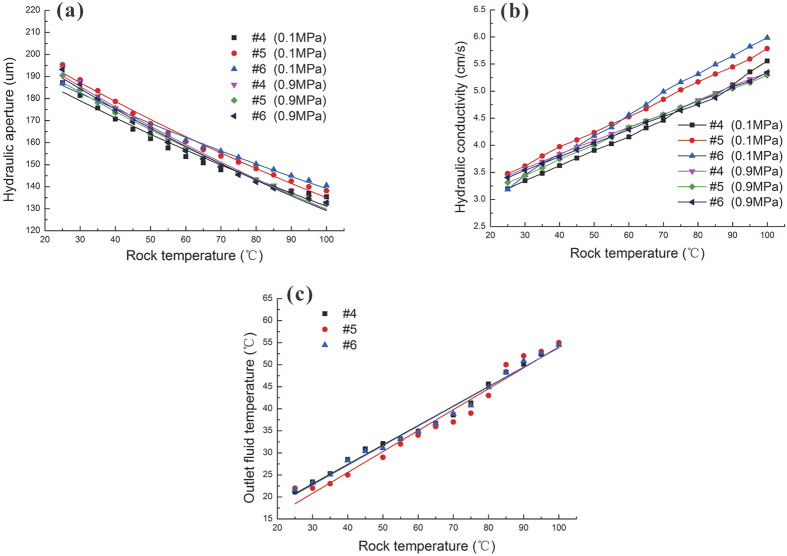
Relationship between rock temperature and hydraulic aperture with the confining pressure is 8.0 MPa (**a**); relationship between rock temperature and hydraulic conductivity with the confining pressure is 8.0 MPa (**b**); and linear relationship between rock temperature and outlet fluid temperature (**c**).

Figure 7a shows the relationship between the rock temperature and the estimated fracture aperture. The fracture aperture decreased with increasing rock temperature. As described in Equation (1), the fracture aperture is determined mainly by the fluid flow rate. Fracture aperture is also determined by the thermal expansion of the granite and the coefficient of thermal expansion (CTE) of granite, which is approximately 3.0×10^−6^ m/°C^16^. Fracture aperture decreased with increasing rock temperature owing to thermal expansion effects. There was a perfect exponential relationship between rock temperature and fracture aperture, as shown in Table 5.

Furthermore, hydraulic conductivity was studied at varying rock temperatures, and the results are shown in Fig. 7b. Sample set two (#4, #5 and 6#) was tested with two different hydraulic pressures (0.1 and 0.9 MPa). In contrast to fracture aperture, hydraulic conductivity increased with increasing rock temperature. As shown in Equation (2), hydraulic conductivity is proportional to fracture aperture and varies inversely with viscosity. The fracture aperture decreased by 46.15% from 190 μm to 130 μm as the temperature increased by 80 °C. The fracture is decreased by 21.16% by calculating the square root. By contrast, it has been previously reported that water viscosity decreases from 100.5 × 10^−5^ Pa·s to 28.38 × 10^−5^ Pa·s when the temperature increases from 20 °C to 100 °C^39^,^40^. Consequently, hydraulic conductivity is calculated to be increased with increasing rock temperature, as described in Equation (2).

### Analysis of heat transfer

Figure 7c shows the outlet fluid temperature and the rock temperature. The outlet fluid temperature increased with increasing rock temperature. A linear relationship was found between these two parameters. This finding indicates that the thermal exchange rate could be enhanced drastically at higher rock temperatures.

**Figure 8:**
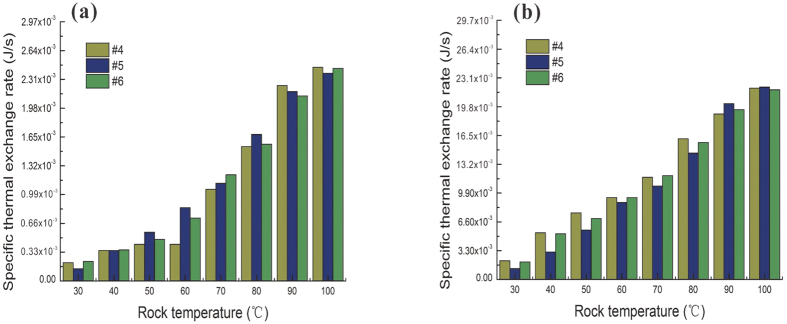
Relationship between rock temperature and heat transfer rate, (**a**) 0.1 MPa hydraulic pressure and 8 MPa confining pressure, (**b**) 0.9 MPa hydraulic pressure and 8 MPa confining pressure.

Calculation of the specific heat-transfer rate was conducted using Equation (3), and the results are presented in Fig. 8. The Equation (3) considers heat convection between fluid and solid rock, represented by the thermal resistance and temperature are different. Three rock samples (#4, #5 and #6) were exposed to a constant confining pressure and two different hydraulic pressures. For both samples, a rising trend is shown between the rock temperature and the specific thermal exchange rate. This finding indicates that thermal efficiency in geothermal exploration could be enhanced drastically at higher rock temperatures. By comparison, the thermal exchange rate changed only slightly when the hydraulic pressure increased from 0.1 MPa to 0.9 MPa, as shown in Fig. 8. This negligible difference could be attributed to the fact that the hydraulic pressure was lower than the confining pressure (8.0 MPa). This finding also implies that hydraulic seepage of fractured samples could remain constant when the confining pressure is more than eight-times larger than the hydraulic pressure.

## Conclusions

In this work, the morphological characteristics of fractures were studied in the two sets of granite samples. The hydraulic properties and the heat-transfer efficiency of these samples were then investigated by coupling different confining pressure and temperature conditions. The influence of confining pressure and temperature on fracture aperture and hydraulic conductivity are discussed and analysed above. The major conclusions from this study are listed below:

1. The morphology of the fractures was determined by 3D modelling of the fracture surface. The area ratio describes the surface roughness by considering the difference between the real surface and projected surface, and was analysed for six rock samples. The area ratio was found to vary widely for different samples. The height distribution frequency of the fractures was also studied. The analysis provided information about the morphological roughness of the fractures that was used for further analysis of the hydraulic properties of the fractured granite samples.
2. The fracture apertures were investigated with a focus on the roughness of the fracture surface. The results show that hydraulic properties are affected mainly by fracture morphology. The area ratio was used as a parameter to describe the roughness as an estimation of hydraulic properties. The larger the area ratio, the larger the fracture aperture, resulting in a higher hydraulic conductivity. This relationship can be attributed to the roughness of the fractures. The ridged parts can easily be destroyed when fitting two pieces of rough rock together as the result of confining pressure. This process leaves small fissures in the rock that fluid can pass through. The higher the degree of roughness, the larger the possibility of creating fissures.
3. The effects of confining pressure: increasing the confining pressure led to a decrease in the size of the fracture apertures. The decreasing trend became small when the confining pressure reached 14.0 MPa. Different rock samples had different decreasing trends of hydraulic conductivity when exposed to increasing confining pressures. This implies that for a fracture with a rougher surface, the hydraulic properties change more drastically as the confining pressure changes.
4. Effects of temperature: the fracture aperture decreased with increasing rock temperature, and a perfectly exponential relationship was observed. The hydraulic conductivity was found to be increased by increasing rock temperature as a result of the drastic decrease in fluid viscosity with increasing temperature. Finally, analysis of the heat transfer of seepage showed that the heat-transfer rate positively correlates with rock temperature, indicating that thermal efficiency could be improved at high rock temperatures.

## Additional Information

**How to cite this article**: Luo, J. *et al*. Experimental investigation of the hydraulic and heat-transfer properties of artificially fractured granite. *Sci. Rep.* **7**, 39882; doi: 10.1038/srep39882 (2017).

**Publisher's note:** Springer Nature remains neutral with regard to jurisdictional claims in published maps and institutional affiliations.

## Figures and Tables

**Table 1:** Basic thermo-physical information of the six rock samples.

Set	No.	Diameter mm	Length mm	Density g/cm^3^	Thermal Conductivity W/m·K	Specific Heat MJ/m^3^·K	Thermal Diffusivity 10^−6^ m^2^/s
One	#1	25.12	31.37	2.71	2.20	1.76	1.25
	#2	25.26	57.62	2.58	2.86	1.93	1.48
	#3	25.26	48.02	2.58	2.86	1.93	1.48
Two	#4	25.06	50.02	2.65	2.28	1.80	1.27
	#5	25.02	50.06	2.65	2.28	1.80	1.27
	#6	25.06	50.02	2.65	2.28	1.80	1.27

**Table 2:** Morphological information of fracture surface of the six rock samples.

Set	No.	Area ratio (−)
No. 1	#1	2.045
	#2	3.344
	#3	4.048
No. 2	#4	4.394
	#5	3.818
	#6	4.341

**Table 3:** Distribution frequency of points’ height of the fractures.

Set	No.	Maximum frequency (%)	Height range (mm)	Secondary frequency (%)	Height range (mm)	Median (mm)
One	#1	4.470	3.454–3.518	4.300	3.518–3.582	3.554
	#2	4.670	5.151–5.258	4.530	6.332–6.439	6.322
	#3	3.400	3.416–3.475	3.400	3.593–3.652	3.182
Two	#4	5.970	3.503–3.577	5.700	3.577–3.652	3.591
	#5	3.050	2.961–3.026	3.000	1.931–1.996	2.257
	#6	3.650	2.030–2.082	3.610	2.082–2.134	2.438

**Table 4:** Hydraulic parameters of the samples set number one.

No.	Temperature (°C)	Confining pressure (MPa)	Fracture aperture (μm)	Hydraulic conductivity (cm/s)
#1	25	4	180	3
#2	25	4	280	7
#3	25	4	425	16

**Table 5:** Exponential fitting of the rock temperature and fracture aperture.

No.	Confining pressure (MPa)	Hydraulic pressure (MPa)	Exponential fitting	R^2^
#4	8	0.1	y = 204.4796e^−0.004x^	0.978
	8	0.9	y = 216.231e^−0.005x^	0.989
#5	8	0.1	y = 215.372e^−0.005x^	0.989
	8	0.9	y = 211.787e^−0.005x^	0.993
#6	8	0.1	y = 204.709e^−0.004x^	0.993
	8	0.9	y = 214.873e^−0.005x^	0.987
